# Supplementary material for: Sox17 and β-catenin co-occupy Wnt-responsive enhancers to govern the endoderm gene regulatory network
Source: eLife. 2020 Sep 7;9:e58029. doi: 10.7554/eLife.58029 (PMC7498262; doi:10.7554/eLife.58029)
Supplement: Supplementary file 6. — Sequence of wild-type and Sox/Tcf mutated enhancers used for luciferase assays. [file elife-58029-supp6.docx]

**Yellow – Sox17**

**Red– Tcf**

1. **-*1kb WT* *six1* enhancer: Chr08:82158915-82159487**

>wt_six1_TSS

AGTGGTTGGTACAACTGAGATCCCTTCCACTACACCTACTTATAACAGGGGAAAGGGGAATTTGATAGGGAGCAATATTGTTCTCTGTCCCATTGGCTGTGGGCACACGTCTGGCCAACTGTGTCTAATTAATGCGAATATCCTTTATTAGGTCATGTGATCATTCTTACCTGATAGCCAAAGTGTATGAAATGACTAGTCAGTCAGTGCCTGAGGATTGCTTTCCCACCTCTCTGTTCCCTTATCTGAACCTATTGTCTTATCTCTGAGCCTCCAGTGCTACCAACAATACATTATCTGTGTTTACAAAGCTAATTGTTTGGGAAATCTTTTTAATAATTGCTTTCAAACAAGGTGTGCAAATGAGGATCAACTTAATTAGAGTGGGACTTCTCTGGGCAATTTTACTAAATACCCTCTTCAAATAAACGAAATATCATGCCTCAGAGTGTTTTCTTTCAGCCTTGCCATTCTCTCTTCAATTAGCCAAGTATTTCCCCACACCCACGGGCCATTATTTCCTCTGGATTCCTGCAAAAGAGGGCAAATTATACGGCTTAACAAAACAAA

1. **-*1kb* ΔSox17 *six1* dSox17: Chr08:82158915-82159487**

>dSox17_six1

AGTGGTTGGTACAACTGAGATCCCTTCCACTACACCTACTTATAACAGGGGAAAGGGGAATTTGATAGGGAGCAATCGCGATCTCTGTCCCATTGGCTGTGGGCACACGTCTGGCCAACTGTGTCTAATTAATGCGAATATCCTTTATTAGGTCATGTGATCATTCTTACCTGATAGCCAAAGTGTATGAAATGACTAGTCAGTCAGTGCCTGAGGATTGCTTTCCCACCTCTCTGTTCCCTTATCTGACCGCGCATCTTATCTCTGAGCCTCCAGTGCTACCAACAATACATTATCTGTGTTTACAAAGCCCGCGCATTTGGGAAATCTTTTTAATAATTGCTTTCAAACAAGGTGTGCAAATGAGGATCAACTTAATTAGAGTGGGACTTCTCTGGGCAATTTTACTAAATACCCTCTTCAAATAAACGAAATATCATGCCTCAGAGTGTTTTCTTTCAGCCTTGCCATTCTCTCTTCAATTAGCCAAGTATTTCCCCACACCCACGGGCCATTATTTCCTCTGGATTCCTGCAAAAGAGGGCAAATTATACGGCTTAACAAAACAAA

1. **Human *-1kb SIX1* enhancer: chr14:61117408-61117704**

>-1kb_six1_hg19

TCATCTGCTGTTTTGTTTATGTGTGGAGAATGCTTAGTAAAACTGCACAAACAAGTGAAGTTGTAATTATCTTAAGCCTCATTTGCACACCTAGTTCCAAAGCAATAATTAAAAGGATTTTGAAAACAATTAGCCTCTCTAAACAAAGATAATCTATTGTCAGTAGCTGAGACTAGCAGGAGATAAGAACAATGCGTGGAGATAAGGGAAGCCAAATGGTGGAAGGAGAGTGACTTTATGACTATACTTATCTCTGATTAGATAAGTTCAAAACAGAATTCTAAGTACACTTCAAAA

1. ***-1.6kb* WT *dkk1_*enhancer: Chr07:5571875-557227**

>wt_dkk1_-2kb

TGAGACCCCCTGGCACAACAACTGATCTATCTATGAAGTCTCCCCCTGACCAATGCCCCCATACATGCACAGAGTGATCAATGCACCTGAGGAAGCAAGATGGCGGCCACAGTAATCACTTGGGCACTGAGTATTTATCACTGCAGCCAATCAAGATGCGAGGAGCTAATGACCAGCGGCCCATTATCACCATCTGATTGTGCCTGTGTCTGGGCTGAGGCCGTTAGAAGGGGGGGACACAGAAGCCCGACACCTTTTGTTCAGTGCAGCCACTAATTGATCTTGGCTAATATAATCAATATTTGATGATCCCGAATCCTTTTCTGCCCCAGACACCTTTTTTATTTGTTATGTGTCcgtacatttacttcatctcttgttttattcttcatatatatag

1. ***1.6kb* ΔSox17 *dkk1_*enhancer (-2kb): Chr07:5571875-557227**

>dSox17_dkk1_-2kb

TGAGACCCCCTGGCTGGATCCTCGATCTATCTATGAAGTCTCCCCCTGACCAATGCCCCCATACATGCACAGAGTGATCAATGCACCTGAGGAAGCAAGATGGCGGCCACAGTAATCACTTGGGCACTGAGTATTTATCACTGCAGCCAATCAAGATGCGAGGAGCTAATGACCAGCGGCCCATTATCCGTATCTAATCTCAGGAGTGTCTGGGCTGAGGCCGTTAGAAGGGGGGGACACAGAAGCCCGACACCTTTTGTTCAGTGCAGCCACTCTTCGGTCTTGGCTAATATAATCAATATTTGATGATCCCGAATCCTTTTCTGCCCCAGACACCTTTTTTATTTGTTATGTGTCcgtacatttacttcagatgacgtcactttcttcatatatatag

1. ***1.6kb* ΔTcf *dkk1_*enhancer: Chr07:5571875-557227**

>dTcf_dkk1_-2kb

TGAGACCCCCTGGCACAACAACTGATCTATATAGTCCAGCTCCCCCTGACCAATGCCCCCATACATGCACAGAGGAGATCTACCACCTGAGGAAGCAAGATGGCGGCCACAGTAATCACTTGGGCACTGAGTATTTATCACTGCAGCCAATCAAGATGCGAGGAGCTAATGACCAGCGGCCCATTATCACCATCTGATTGTGCCTGTGTCTGGGCTGAGGCCGTTAGAAGGGGGGGACACAGAAGCCCGACACCGCCTACGATATGCAGCCACTACTACTCTGTGGCTAATATAATCAATGCCATCACTTCCCGAATCCTTTTCTGCCCCAGACACCTTTTTTATTTGTTATGTGTCcgtacatttacttcatctcttgttttattcttcatatatatag

1. ***-7.5*kb WT *dkk1* enhancer: chr7:5,565,983-5,566,438**

>wt_dkk1 -7.5kb

AGCCGAGCTGCTTGTGATCTCCTTCCCTCCGCTATCAGCATCTCTATTCATTCTGGCTTCAAAGCCGCGGCTTCCCCACACTCATGCCCCTTTCTTCCTCGCCGGTTAGTAATGAAGTTAGGAGCGTGTGTGTTTGCTCGTGCAAACGACATTGTAAGGAAATAGCGGCTATTACAGGCTGAGTGGATTTAGGGAGGCAGAAGCCTTAGAAGTAGGGGGGGGGATTACTCGACCGCTTAACTAGATGAATGTGCTGAGCACAATGTCTGCGCGCCCAAAAGACAACTGAGAAAAGCCGAGCAAACGGACTATTTGTAAAAAGTGGCATCTTTGCTAATGTCTGATTAAAAGCCGGGGTCCCGGGGCTGTTTGATCTGCGCTCATCAAAGGGAGCCTTAAACAAGCTTGGTTTAAAGCCGCTGAAAGGCCTCAGCGCGGGCTGAGAAAACAAAGGCC

1. ***-7.5*kb ΔSox17 *dkk1* enhancer: chr7:5,565,983-5,566,438**

>sox17_dkk1 -7.5kb

AGCCGAGCTGCTTGTGATCTCCTTCCCTCCGCTATCAGCATCTCTATTCATTCTGGCTTCAAAGCCGCGGCTTCCCCACACTCATGCCCCTTTCTTCCTCGCCGGTTAGTAATGAAGTTAGGAGCGTGTGTGTTTGCTCGTGCAAACGACATTGTAAGGAAATAGCGGCTATTACAGGCTGAGTGGATTTAGGGAGGCAGAAGCCTTAGAAGTAGGGGGGGGGATTACTCGACCGCTTAACTAGATGAATGTGCTGAGCGTCCGATCTGCGCGCCCAAAAGACAACTGAGAAAAGCCGAGCAAACGGACGTCCACTAAAAAGTGGCATCTTTGCTAATGTCTGATTAAAAGCCGGGGTCCCGGGGCTGTTTGATCTGCGCTCATCAAAGGGAGCCTTAAAGTCGATTGGTTTAAAGCCGCTGAAAGGCCTCAGCGCGGGCTGAGAAAACAAAGGCC

1. ***-7.5*kb** Δ**Tcf *dkk1* enhancer: chr7:5,565,983-5,566,438**

>sox17_dkk1_-7.5kb

AGCCGAGCTGCTTGTGATCTCCTTCCCTCCGCTATCAGCATCTCTATTCATTCTGGCTTTGCCGCCGCGGCTTCCCCACACTCATGCCCCTTTCTTCCTCGCCGGTTAGTAATGAAGTTAGGAGCGTGTGTGTTTGCTCGTGCAAACGACATTGTAAGGAAATAGCGGCTATTACAGGCTGAGTGGATTTAGGGAGGCAGAAGCCTTAGAAGTAGGGGGGGGGATTACTCGACCGCTTAACTAGAGACCGATGCTGAGCGTCCGATCTGCGCGCCCAAAAGACAACTGAGAAAAGCCGAGCAAACGGACGTCCACTAAAAAGTGGCATCTTTGCTAATGTCTGATTAAAAGCCGGGGTCCCGGGGCTGTTTGATCTGCGCTCATTGCCGGGAGCCTTAAAGTCGATTGGTTTAAAGCCGCTGAAAGGCCTCAGCGCGGGCTGAGAAAACAAAGGCC

1. **TSS WT *lhx5_*enhancer: Chr01:133702140-133702540**

>wt_lhx5_tss

GGGTTACTGGCAGGATTGGTTTGGGCTTTGCTTTACAAAGAAACAGGAGGGGGGTCTGGAATTTTGTTTTCACCTAATCCAGATTTTGTATGTGAATAGGACAGTAGGAGGGAATTCCCTGTAGGACTCCCTAACCACCCCAAACACCAAGGTAAATAAACTTAGCATTAAGCAAACAATTAATACACTGCCCTTTCATAGAACTTCATTATGATGCTGTTTTCTCCACCCAGTTAACCTCTCTATAACTGAATCTGCATGTGAAACACCTAGCTCGTTAGCACTGCAGGGGTTAAACGGGTTTGCCTGTTTCTCAGTACAGCTAGCACACTTACTGATACAAAGTGTCACATACGACTCATAAGTGGCAAACAGAATAATACTGGGGTTATTTTAAGGG

1. **TSS ΔSox17 *lhx5_*enhancer: Chr01:133702140-133702540**

>dSox17_lhx5

GGGTTACTGGCAGGATTGGTTTGGGCCGTATCTATACTTCACACAGGAGGGGGGTCTGGAATCGAACGCGCACCTAATCCAGATTTTGACTTCCTACAGGACAGTAGGAGGGAATTCCCTGTAGGACTCCCTAACCACCCCTATGTCGAAGGTAAATAAACTTAGCATTAAGCATGTGGCACCTACACTGCCCTTTCATAGAACTTCATTATGATGCTGTTTTCTCCACCCAGTTAACCTCTCTACCGGCATGTCTGCATGTGAAACACCTAGCTCGTTAGCACTGCAGGGGTTAAACGGGTTTGCCTGTTTCTCAGTACAGCTAGCACACTTACTGATACAAAGTGTCACATACGACTCATAAGTGGCAAGGCTAGCGCTACTGGGGTTATTTTAAGGG

1. **TSS ΔTcf *lhx5_*enhancer: Chr01:133702140-133702540**

>dTcf_lhx5

GGGTTACTGGCAGGATTGGTCAGAGTAACCGTGCAGAAAGAAACAGGAGGGGGGTCTGGAATTTTGTTTTCACCTAATCCAGATTTTGTATGTGAATAGGACAGTAGGAGGGAATTCCCTGTAGGACTCCCTAACCACCCCAAACACCAAGGTAAATAAACTTAGCATTAAGCAAACAATTAATACACTTACCATGACTAGAACTTCATTATGATGCTGTTTTCTCCACCCAGTTAACCTCTCTATAACTGAATCTGCATGTGAAACACCTAGCTCGTTAGCACTGCAGGGGTTAAACGGGTTTGCCTGTTTCTCAGTACAGCTAGCACACTTACTGATACAAAGTGTCACATACGACTCATAAGTGGCAAACAGAATAATACTGGGGTTATTTTAAGGG

1. ***WT* +7kb_*osr1*: Chr05: 138520826- 138520976**

>wt_osr1

AATCGGCTGCTCATGTCTGTGCATTGCTGCTTCTGTAGAATTGTATTTCTgtacgcgtccaaatgaattgctaattaaccttaatctgaattgcatgtgtctggctttatagggttgatgctgcTAATTAATCAGCTTGTTTGGACATGT

1. *Δ****Sox17* +7kb_*osr1*: Chr05: 138520826- 138520976**

>dSox17_osr1

AATCGGCTGCTCATGTCTGTGCATTGCTGCTTCCAGTTACTTAGGACTCTgtacgcgtcagactgttatgctaattaaccttcagaggtgatgcatgtgtctggctttatagggttgatgctgcTAATTAATCAGCTGACAAGGACATGT

1. ***WT* +3kb_*igf3*: Chr07: 103116558- 103116708**

>wt_igf3

CAAACACCTAGGAACCATGCAGCGATTGCTGGAAAGAGACAGACAATGTAATACATTGGATTGTGCACCACTTAATTGTATGGATAATAGGTGTCACATCTGAGTTACACAAGCAATTGAAAGCACAAGGCTGCAAGTCAAGGTAAATTA

1. *Δ****Sox17* +3kb_*igf3*: Chr07: 103116558- 103116708**

>dSox17_igf3

CAAACACCTAGGAACCATGCAGCGATTGCTGGAAAGAGGCACTGTTAGTAATACATTGGATTCAGTTACACTTACGGGCTGAGATAATAGGTGTCACATCTGAGTTACACAAGCAATTGATAGGACAAGGCTGCAAGTCAAGGTAAATTA
