# Supplementary material for: Sox17 and β-catenin co-occupy Wnt-responsive enhancers to govern the endoderm gene regulatory network
Source: eLife. 2020 Sep 7;9:e58029. doi: 10.7554/eLife.58029 (PMC7498262; doi:10.7554/eLife.58029)
Supplement: Transparent reporting form [file elife-58029-transrepform.pdf]

eLIFE
Westbrook Centre, Milton Road
Cambridge CB4 1YG
UK
P 01223 855340
W elifesciences.org
T @elife

eLife's transparent reporting form

We encourage authors to provide detailed information *within their submission* to facilitate the interpretation and replication of experiments. Authors can upload supporting documentation to indicate the use of appropriate reporting guidelines for health-related research (see EQUATOR Network), life science research (see the BioSharing Information Resource), or the ARRIVE guidelines for reporting work involving animal research. Where applicable, authors should refer to any relevant reporting standards documents in this form.

If you have any questions, please consult our Journal Policies and/or contact us: editorial@elifesciences.org.

Sample-size estimation

- You should state whether an appropriate sample size was computed when the study was being designed
- You should state the statistical method of sample size computation and any required assumptions
- If no explicit power analysis was used, you should describe how you decided what sample (replicate) size (number) to use

Please outline where this information can be found within the submission (e.g., sections or figure legends), or explain why this information doesn't apply to your submission:

No power calculation was performed, and sample size was based on the largest practical for embryonic material. RNA-Seq and ChIP-Seq was performed on a time course of sibling control and experimental embryos in biological duplicates. Ten embryos were pooled for each time point. Separate embryo batches were used for duplicates. More details can be found in the method section: '*RNA-Sequencing: RNA isolation and library preparation*'.

Replicates

- You should report how often each experiment was performed
- You should include a definition of biological versus technical replication
- The data obtained should be provided and sufficient information should be provided to indicate the number of independent biological and/or technical replicates
- If you encountered any outliers, you should describe how these were handled
- Criteria for exclusion/inclusion of data should be clearly stated
- High-throughput sequence data should be uploaded before submission, with a private link for reviewers provided (these are available from both GEO and ArrayExpress)

Please outline where this information can be found within the submission (e.g., sections or figure legends), or explain why this information doesn't apply to your submission:

- Genomic experiments performed at least twice, other experiment more that three often 4-6 times.
- Detailed information about number of embryos and replicates used per experiment can be found in the methods and figures.

- Sequencing data has been uploaded to GEO and accession ID GSE148726 has been provided in the manuscript '*Data and Software Availability*' section. The reviewer access link is https://www.ncbi.nlm.nih.gov/geo/query/acc.cgi?acc=GSE148726 and the token is choluuuefpkprsb
- We did not encounter outliers.

Statistical reporting

- Statistical analysis methods should be described and justified
- Raw data should be presented in figures whenever informative to do so (typically when N per group is less than 10)
- For each experiment, you should identify the statistical tests used, exact values of N, definitions of center, methods of multiple test correction, and dispersion and precision measures (e.g., mean, median, SD, SEM, confidence intervals; and, for the major substantive results, a measure of effect size (e.g., Pearson's r, Cohen's d)
- Report exact p-values wherever possible alongside the summary statistics and 95% confidence intervals. These should be reported for all key questions and not only when the p-value is less than 0.05.

Please outline where this information can be found within the submission (e.g., sections or figure legends), or explain why this information doesn't apply to your submission:

- Details of the statistical test used for each analysis can be found in the methods and figure legends.
- For luciferase reporter assays raw data points are presented in the figures and for in situ hybridization the number of embryos is indicated.
- ChIP-qPCR experiments: experiments were repeated at least thrice and representative enrichment was reported.

(For large datasets, or papers with a very large number of statistical tests, you may upload a single table file with tests, Ns, etc., with reference to sections in the manuscript.)

Group allocation

- Indicate how samples were allocated into experimental groups (in the case of clinical studies, please specify allocation to treatment method); if randomization was used, please also state if restricted randomization was applied
- Indicate if masking was used during group allocation, data collection and/or data analysis

Please outline where this information can be found within the submission (e.g., sections or figure legends), or explain why this information doesn't apply to your submission:

N/A

Additional data files ("source data")

- We encourage you to upload relevant additional data files, such as numerical data that are represented as a graph in a figure, or as a summary table
- Where provided, these should be in the most useful format, and they can be uploaded as "Source data" files linked to a main figure or table
- Include model definition files including the full list of parameters used
- Include code used for data analysis (e.g., R, MatLab)
- Avoid stating that data files are "available upon request"

Please indicate the figures or tables for which source data files have been provided:

- Fig 1D, E; Fig S1A, E – Underlying data can be found at Supplementary Table S1
- Fig 2, Fig S3 – Supplementary Table S1, S2.
- Fig 3, Fig S5 – Supplementary Table S3, S4
- Fig 4 A-B Fig S6 A, C, D: Supplementary Tables S1 – S4
- Fig 4C, E: Supplementary Table S5
- Fig 6, S8, S9: Supplementary File S1: Supp_luc_enhancer_sequences.docx
- Additionally, all raw sequencing data has been uploaded to GEO and information can be found in the '*Data and Software Availability*' section.
